# BCR/ABL1 and BCR are under the transcriptional control of the MYC oncogene

**DOI:** 10.1186/s12943-015-0407-0

**Published:** 2015-07-16

**Authors:** Nitesh Sharma, Vera Magistroni, Rocco Piazza, Stefania Citterio, Caterina Mezzatesta, Praveen Khandelwal, Alessandra Pirola, Carlo Gambacorti-Passerini

**Affiliations:** Department of Health Sciences, University of Milano Bicocca, Monza, Italy; Division of Haematology, San Gerardo Hospital, Monza, Italy; Department of Bioscience and Biotechnology, University of Milano Bicocca, Milano, Italy

**Keywords:** CML, BCR/ABL1, MYC

## Abstract

**Background:**

Chronic Myeloid Leukaemia (CML) is caused by the BCR/ABL1 fusion gene. Both the presence and the levels of BCR/ABL1 expression seem to be critical for CML progression from chronic phase (CP) to blast crisis (BC). After the oncogenic translocation, the BCR/ABL1 gene is under the transcriptional control of BCR promoter but the molecular mechanisms involved in the regulation of oncogene expression are mostly unknown.

**Methods:**

A region of 1443bp of the functional BCR promoter was studied for transcription factor binding sites through in-silico analysis and Chromatin Immunoprecipitation experiments. BCR and BCR/ABL1 expression levels were analysed in CML cell lines after over-expression or silencing of MYC transcription factor. A luciferase reporter assay was used to confirm its activity on BCR promoter.

**Results:**

In the present study we demonstrate that MYC and its partner MAX bind to the BCR promoter, leading to up-regulation of BCR and BCR/ABL1 at both transcriptional and protein levels. Accordingly, silencing of MYC expression in various BCR/ABL1 positive cell lines causes significant downregulation of BCR and BCR/ABL1, which consequently leads to decreased proliferation and induction of cell death.

**Conclusions:**

Here we describe a regulatory pathway modulating BCR and BCR/ABL1 expression, showing that the BCR promoter is under the transcriptional control of the MYC/MAX heterodimer. Since MYC is frequently over-expressed in BC, this phenomenon could play a critical role in BCR/ABL1 up-regulation and blast aggressiveness acquired during CML evolution.

## Background

Chronic Myeloid Leukaemia (CML) is a clonal myeloproliferative disorder caused by the constitutive tyrosine kinase activity of the BCR/ABL1 fusion protein, the product of the Philadelphia (Ph) chromosome, generated from the t(9;22)(q34;q11) translocation [1]. If untreated, CML progresses within 3-5 years from a mild and easy to control form, called chronic phase (CP), to the aggressive and incurable blast crisis (BC), the final phase of this disease. In CP, BCR/ABL1 expression induces a survival advantage but leukemic cells hold their capacity to differentiate normally. Conversely BC, as any acute leukemia, is marked by a complete differentiation block and by the ensuing accumulation of blasts.

At the molecular level BC is a heterogeneous disease [2, 3]. Regardless of the additionally secondary changes, one common feature during the evolution from CP to BC is a marked increase in BCR/ABL1 expression [3]. Progression to BC possibly occurs as a result of this increase, which leads to the activation of several events in primitive progenitor’s cell, such as genomic instability, acquisition of resistance to apoptosis, and activation of beta catenin in granulocyte-macrophage progenitors resulting in the acquisition of self-renewal capacity [4–8]. Although the biological consequences of BCR/ABL1 up-regulation have been intensively studied, the molecular mechanisms responsible for this increase in expression are mostly unexplored.

After the oncogenic translocation, the BCR/ABL1 gene is under the transcriptional control of the BCR promoter, which may play a critical role in controlling BCR/ABL1 expression [9]; in fact a similar dysregulation of both BCR and BCR/ABL1 gene transcription is evident in BC [10]. Limited efforts have been devoted until now to the characterization of BCR promoter: according to Shah et al. [9] a functional promoter is localized in a region of 1Kb at the 5′ of the BCR exon 1 coding sequence. Recent results from Marega et al confirmed the importance of this region for BCR basal transcriptional level: this study showed that the sequence comprised between -1443 and -1202 bp from the ATG site is critical to achieve a high level of *BCR* promoter activity [10].

MYC is a transcription factor belonging to the basic-helix-loop-helix-leucine zipper (bHLH-LZ) family [11]. It forms heterodimers with the bHLH-LZ partner MAX, subsequently binding to a core DNA consensus region. Assembly of MYC/MAX heterodimer and DNA-binding seems to be crucial for the mitogenic, oncogenic and antiapoptotic functions of MYC [12, 13]*.* MYC is activated in various cancers through different genomic events including chromosomal translocation or gene amplification [14–18]. MYC protein has been shown to play a role in BCR/ABL1 mediated transformation, mainly by acting as a cooperative oncogene with the fusion protein [19–21]. Furthermore, BCR/ABL1 can induce MYC activity through distinct mechanisms: by regulating MYC expression through PI3K, JAK2 pathways and the E2F1 transcription factor [22–24], by inhibiting MYC proteasome-dependent degradation through activated JAK2 [25] and by regulating MYC mRNA translation by enhancing HNRPK translation-regulation activity [26]. MYC is also a known BCR binding partner [27] and higher BCR levels can decrease MYC protein levels, thus suggesting that BCR monoallelic disruption through BCR/ABL1 translocation may contribute to MYC protein stability in CML. Interestingly, MYC expression is normal in CP-CML, but is frequently up-regulated in BC through chromosome 8 amplification or over-expression [28]. Recently, Lucas CM et al. [29] showed that pharmacological inhibition of MYC reduces BCR/ABL1 tyrosine kinase activity and its expression level. These data suggest a causative role for MYC in the regulation of BCR/ABL1 expression, but they did not identify the molecular basis of this phenomenon. In the present study we demonstrate for the first time that MYC/MAX heterocomplex binds to the BCR promoter at four specific binding sites, leading to up-regulation of BCR and BCR/ABL1 at both transcriptional and protein levels in CML cell lines. Accordingly, silencing of MYC expression in various BCR/ABL1 positive cell lines causes significant downregulation of BCR and BCR/ABL1, decreases proliferation rate and induces cell death in CML cells. Since MYC is frequently over-expressed in BC, this phenomenon could be responsible for the BCR/ABL1 up-regulation and blast aggressiveness showed during CML evolution.

## Results

### In-silico identification of MYC and MAX transcription factors binding sites on BCR promoter

**Fig. 1:**
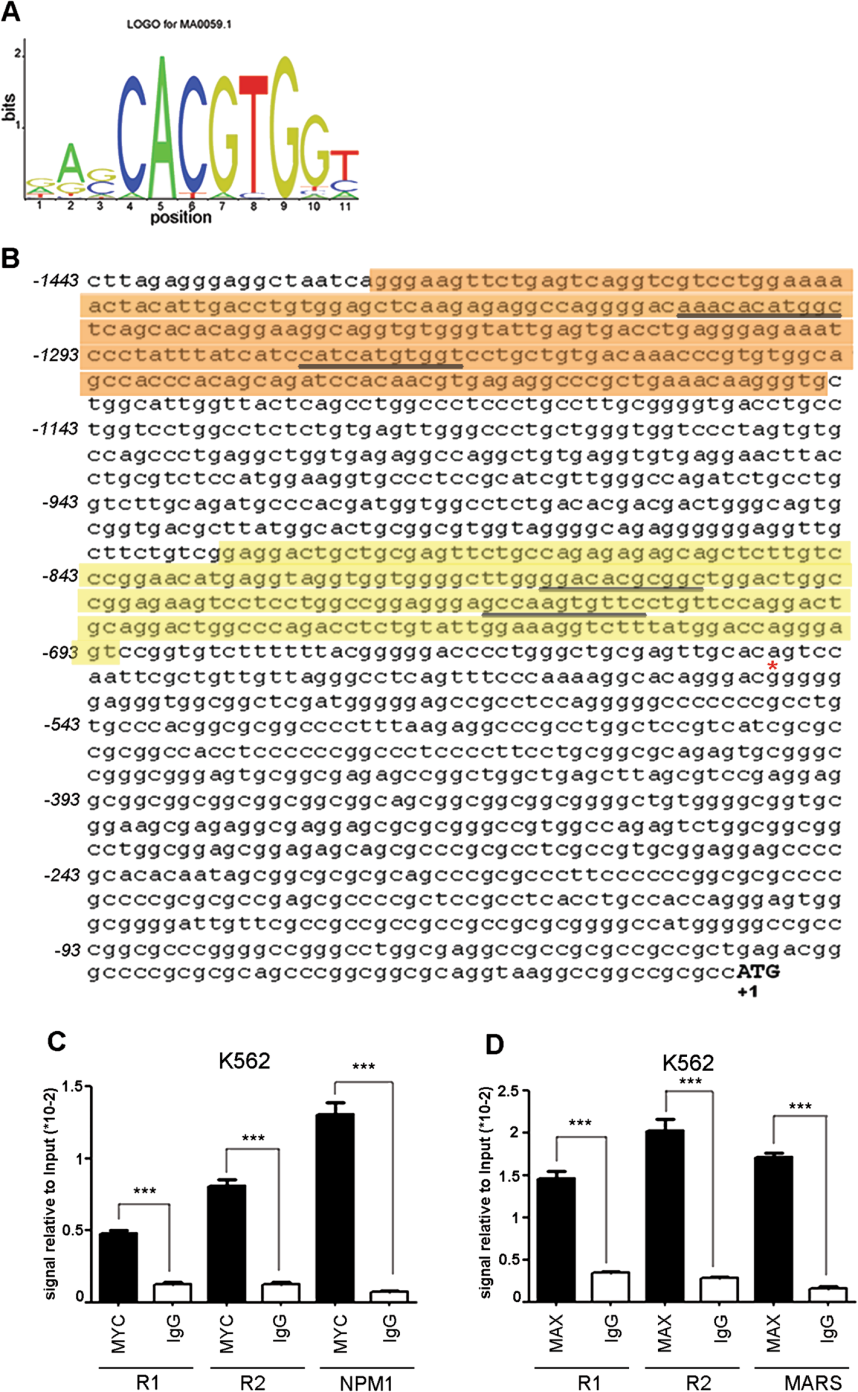
MYC and MAX binding at BCR promoter. (**a)** Schematic representation of 11-nucleotide position frequency matrix for MYC/MAX binding (MA0059.1) as obtained from Jaspar core database. The height of the nucleotides of the “sequence logo” represents the conservation of the nucleotides measured in bits (binary digit) (**b**) Nucleotide sequence of the BCR promoter region analyzed in this study. Lowercase nucleotides represent the regions cloned in the pGL3 vector. Consensus regions for MYC/MAX binding are underlined. The regions amplified in Chromatin Immunoprecipitation (ChIP) analysis are highlighted in orange (Region 1, R1) and yellow (Region 2, R2). The BCR transcriptional starting site, according to NM_021574.2 and NM_004327.3, is highlighted as a red asterisk. (**c**, **d)** ChIP results for MYC and MAX binding at region1 (R1) and region 2 (R2) and for MYC and MAX positive controls. The RTq-PCR data represent the means ± SD (standard deviation) of two independent experiments. An IgG antibody was used as negative control. Signals are normalized to the Input DNA. *** = *p* < 0.0001

To identify the transcription factors directly involved in BCR promoter regulation, we studied a region of 1443bp upstream of the human BCR gene coding sequence (Breakpoint Cluster Region; NC_000022.11). According to Marega et al. [10] and to previous studies [9, 30], this region contains critical elements for the regulation of BCR transcription. Through *in-silico* analysis we identified distinct putative binding sites for several transcription factors [10]. Criteria for selecting the transcription factors (TFs) were: 1) TFs binds to a DNase protected area of *BCR* promoter [9]; 2) TFs have role in hematopoiesis or differentiation of hematopoietic progenitors. Based on the DNA binding matrix shown in Fig. 1a we found four highly relevant MYC/MAX binding sites (PBS) located at PBS1 (-1354 bp to -1364bp), PBS2 (-1279 to -1269 bp), PBS3 (- 813 bp to -803 bp) and PBS4 (-767 bp to -757 bp) (Fig. 1b).

### MYC and MAX bind to BCR promoter

To assess the validity of the *in-silico* prediction, we performed Chromatin Immunoprecipitation analyses (ChIP) on the BCR/ABL1-positive K562 cell line, which showed a highly significant enrichment for BCR promoter on the previously identified regions after MYC and MAX immunoprecipitations compared to the negative control (MYC: MYC_R1 0.0048 ± 0.0002, IgG_R1 0.001 ± 0.00009; MYC_R2 0.01 ± 0.0005, IgG_R2 0.001 ± 0.0002; MAX: MAX_R1 0.01 ± 0.0009, IgG_R1 0.0035 ± 0.0001, MAX_R2 0.02 ± 0.001, IgG_R2 0.0029 ± 0.0002; MYC_R1 vs IgG_R1, *p* < 0.0001; MYC_R2 vs IgG_R2 p < 0.0001; MAX_R1 vs IgG_R1 *p* < 0.0001; MAX_R2 vs IgG_R2 *p* < 0.0001) (Fig. 1c and d). The Nucleophosmin (*NPM1*) [31] and the methionyl-tRNA synthetase (*MARS*) [32] promoters were used as positive controls for MYC and MAX binding, respectively (MYC_NPM1 0.01 ± 0.0008, IgG_NPM1 0.00076 ± 0.00009; MAX_MARS 0.02 ± 0.0006, IgG_MARS 0.0017 ± 0.0002, MYC_NPM1 vs IgG_NPM1 *p* < 0.0001; MAX_MARS vs IgG_MARS *p* < 0.0001).

### Overexpression of MYC and MAX causes BCR and BCR/ABL1 up-regulation in CML cell lines

**Fig. 2:**
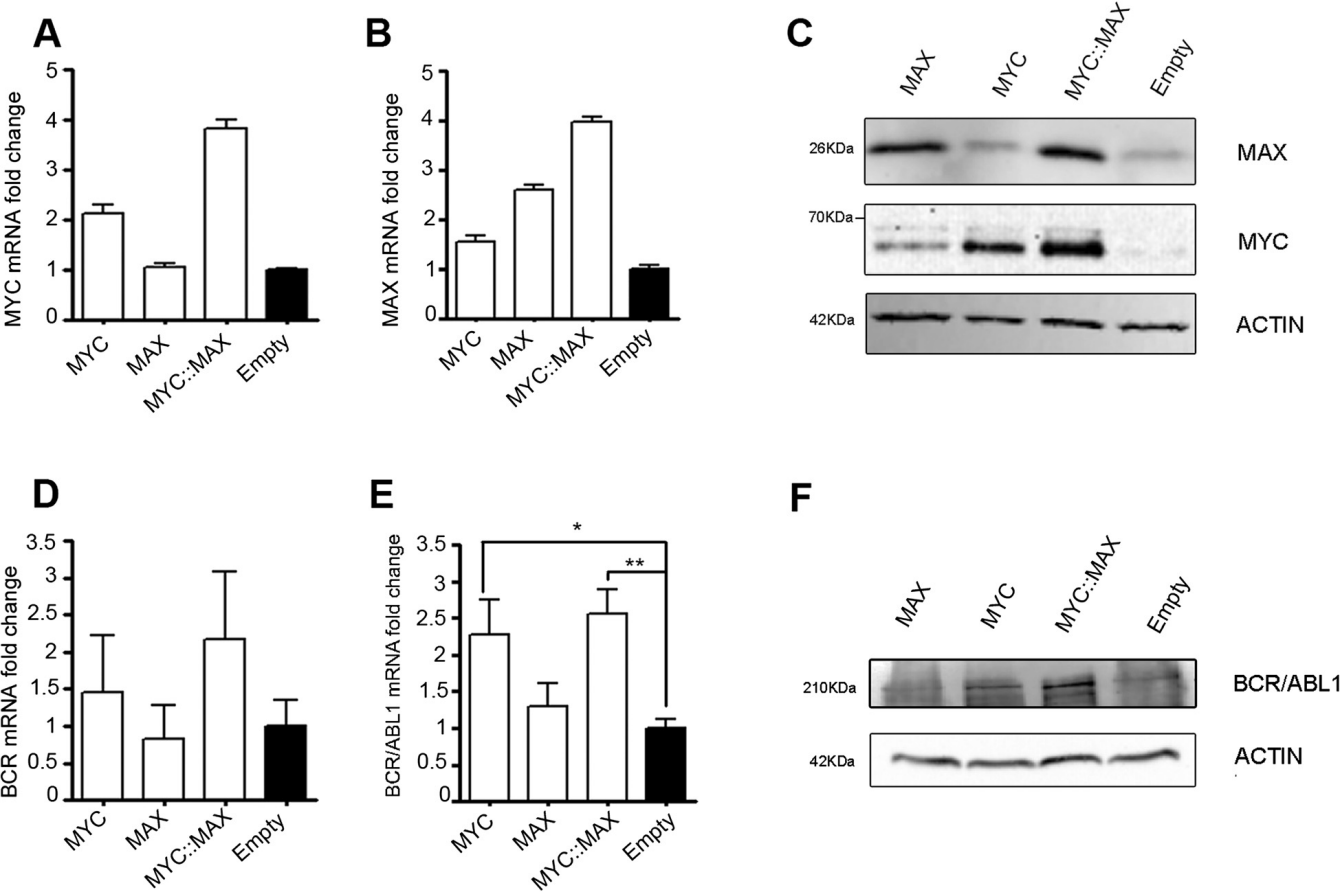
Effect of MYC and MAX on BCR and BCR/ABL1 expression in K562 cell line. MYC (**a,c**) and MAX (**b,c**) over-expression in K562 cells stably transfected with MAX, MYC, or pcDNA3 empty vector (Empty) or MYC/MAX (MYC::MAX), as evidenced by RTq-PCR (**a,b**) and Western Blot from total lysates (**c**). RT-qPCR (**d,e**) and Western Blot (**f**) show BCR and BCR/ABL1 expression levels in K562 transfectants. Actin was used as loading control. The RTq-PCR data shown in (**d,e**) represent the means ± SD (standard deviation) of three independent experiments. Data are represented as mRNA fold change compared to the Empty sample

To investigate the role of MYC and MAX binding on BCR promoter, we overexpressed MYC, MAX and MYC/MAX in the BCR/ABL1-positive K562 cell line (Fig. 2a-c) and we assessed the effect of MYC/MAX overexpression on BCR and BCR/ABL1 at mRNA and protein levels. We found that, both MYC and MYC/MAX over-expression significantly upregulate BCR/ABL1 compared to control cells (Empty), as assessed by Real-Time quantitative PCR (RT-qPCR) (MYC_BCR/ABL1 fold induction: 2.27 ± 0.48, *p* = 0.01; MYC/MAX_BCR/ABL1 fold induction: 2.55 ± 0.34, *p* = 0.002) and western blot analysis. A similar trend is also shown for BCR levels (MYC_BCR fold induction: 1.46 ± 1.34, *p* = 0.62; MYC/MAX_BCR fold induction: 2.17 ± 0.92, *p* = 0.14) (Fig. 2d-f). Interestingly, over-expression of MAX alone is not able to induce significant up-regulation of both BCR and BCR/ABL1 (MAX_BCR fold induction: 0.83 ± 0.79, *p* = 0.78; MAX_BCR/ABL1 fold induction: 1.30 ± 0.32, *p* = 0.2).

### Silencing of MYC causes a significant decrease in BCR and BCR/ABL1 levels and induces cell death in CML cell lines

**Fig. 3:**
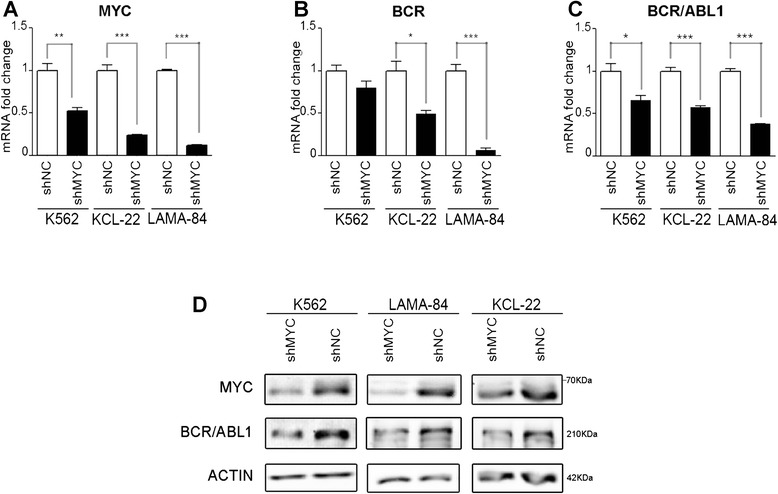
MYC silencing causes downregulation of BCR and BCR/ABL1 expression. The BCR/ABL1-positive cell lines K562, KCL-22 and LAMA-84 were infected with lentiviruses expressing scrambled shRNA (negative control: shNC) or MYC shRNA (shMYC). (**a-c**) RT-qPCR for MYC, BCR and BCR/ABL1 levels. Data are represented as mRNA fold change compared to the shNC sample. ***: *p* < 0.001, **: *p* < 0.01,*: *p* < 0.05. Signals are representative of two independent experiments (**d**) Western Blot analysis of total cell lysates. Actin was used as loading control

To further test the effect of MYC on BCR and BCR/ABL1 expression, we generated a lentiviral silencing model targeting MYC expression in the K562 cell line. The effect of the lentiviral silencing was assessed by mean of RT-qPCR and western blot on BCR/ABL1 and BCR. MYC silencing significantly reduces BCR/ABL1 levels in K562 cells compared to the negative control, as determined by RT-qPCR (BCR/ABL1 fold change: 0.66 ± 0.08, *p* = 0.03) and western blot (Fig. 3a, c and d). A similar trend is observed for BCR level (BCR fold change: 0.8 ± 0.13, *p* = 0.12) (Fig. 3b). The test if the effect of MYC down-modulation on BCR and BCR/ABL1 expression was limited to the K562 cells or was evident on other BCR/ABL1 positive cell lines, we generated MYC silencing models in KCL-22 and LAMA-84, showing significant down-regulation of both BCR and BCR/ABL1 compared to their negative controls at the mRNA (BCR fold change: KCL-22 0.49 ± 0.07, *p* = 0.013; LAMA-84 0.07 ± 0.03, *p* = 0.0003. BCR/ABL1 fold change: KCL-22 0.58 ± 0.02, *p* = 0.0007; LAMA-84 0.38 ± 0.006, *p* < 0.0001) and protein levels (Fig. 3b-d).

**Fig. 4:**
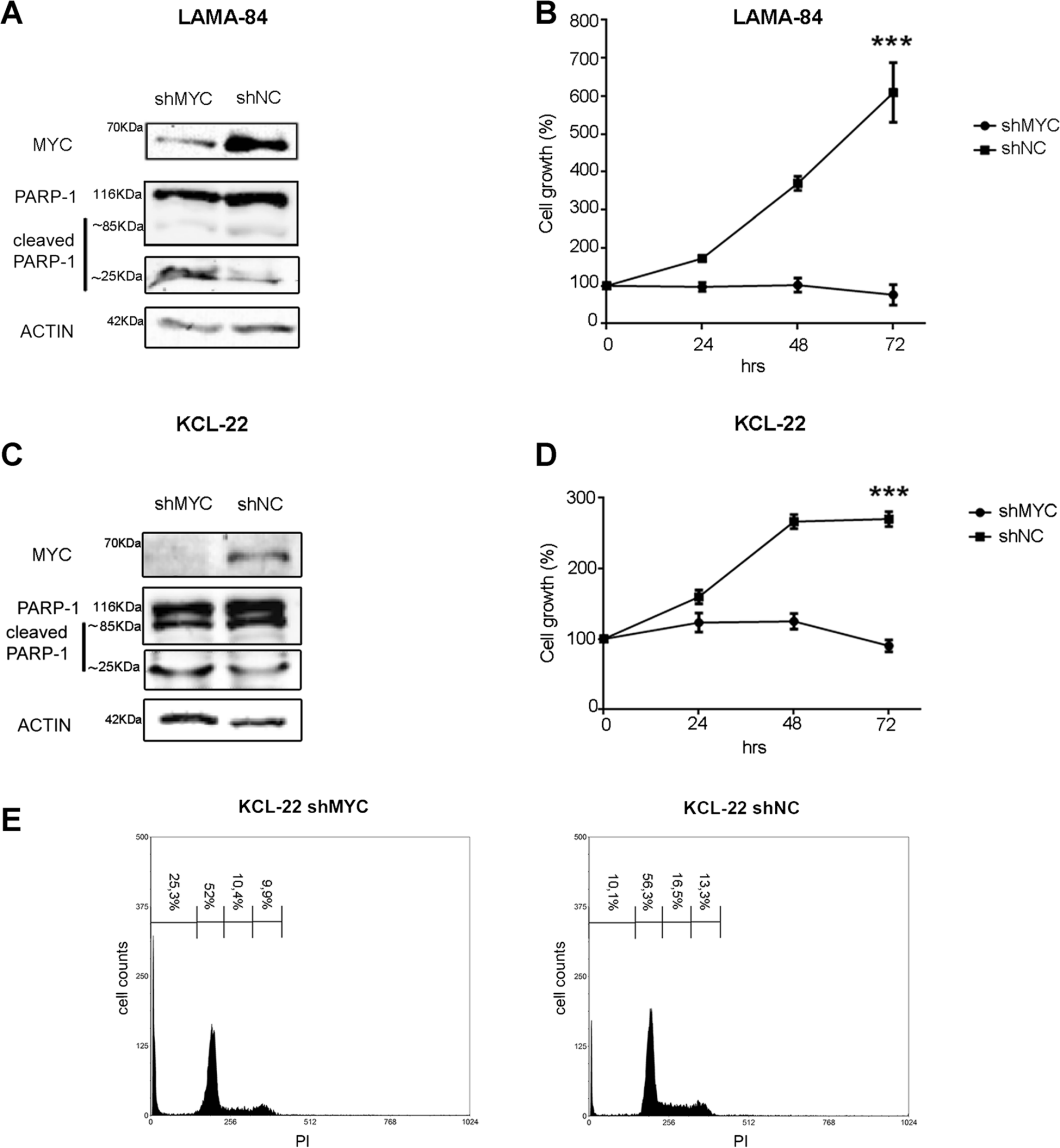
Biological role of MYC silencing in CML cell lines. Immunoblots for MYC, PARP-1 and Actin in LAMA-84 (**a**) and KCL-22 (**c**) cell lines encoding MYC-shRNA (shMYC) or the control shRNA (shNC). The PARP-1 antibody recognizes total (116kDA) and cleaved PARP-1 (85/25kDa). Cell viability of the same cells (**b,d**) was determined by the MTS assay. Values represent the mean normalized percentage of survival compared to control cells (n = 5 wells; ± SD). (***: *p* < 0.001). (**e**) Flow cytometry analysis of propidium iodide (PI) stained KCL-22 cells after 72h of MYC silencing

Finally, given the recognized role of MYC in v-ABL1 transforming potential [21], we confirmed that MYC silencing in CML cell lines resulted, together with BCR and BCR/ABL1 down-modulation, in a highly significant growth arrest (*p* < 0.0001) (Fig. 4b and d) and in induction of cell death (Fig. 4a, c and e).

### MYC controls the activity of BCR promoter

**Fig. 5:**
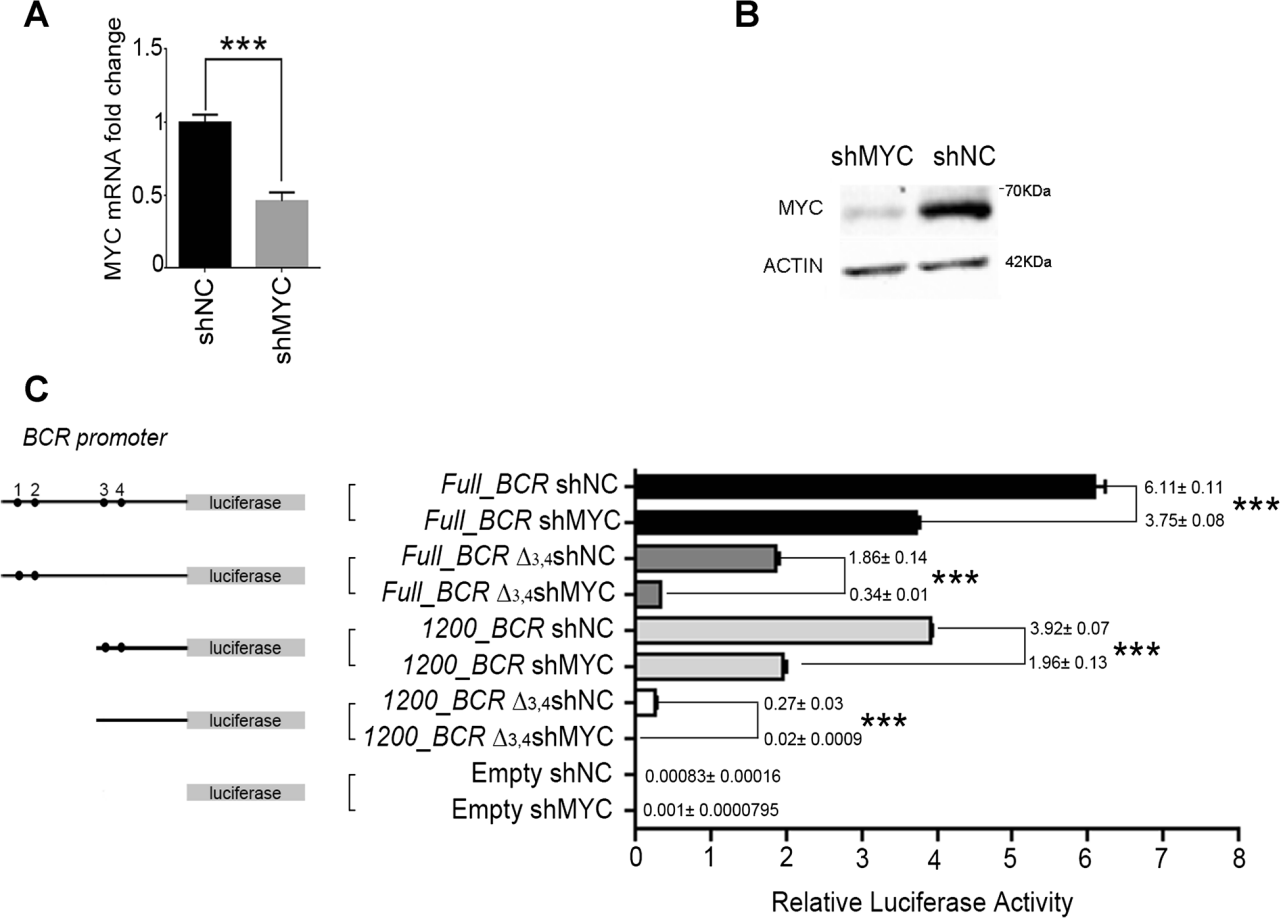
PBS1-4 are critical for BCR promoter regulation. 293 cells were infected with lentiviruses expressing scrambled shRNA (negative control: shNC) or MYC shRNA (shMYC). (**a**,**b**) RT-qPCR and Western Blot analyses assessing MYC expression levels. (**c**) Luciferase assay on 293 infected cells. The four MYC/MAX binding sites are numbered in the figure (1-4). The graph shows the relative luciferase values as assessed after normalization with Renilla Luciferase signal. Values reported in the graph represent the average of three separate experiments. *** = *p* < 0.0001

To define if the modulation of BCR and BCR/ABL1 expression by MYC is a direct consequence of MYC activity on the four binding sites identified on BCR promoter, we set-up a luciferase reporter assay on 293T cells (Fig. 5). We created BCR promoter constructs as follows: 1) Full_BCR: 1443 bp upstream of the BCR coding starting site comprising all four MYC/MAX binding sites, 2) Full_BCR∆34: 1443 bp upstream of the BCR coding starting site with deletion of a region comprising the third and fourth MYC/MAX binding sites, 3) 1200_BCR: 1204 bp upstream of the BCR coding starting site with only the third and fourth MYC/MAX binding sites; 4) 1200_BCR∆34: 1204 bp upstream of the ATG with no MYC/MAX binding sites (Fig. 5c). In order to assess the effect of MYC/MAX on BCR promoter we tested the activity of the luciferase reporter assay in presence of MYC expression and in a lentiviral MYC silencing model (Fig. 5a and b), showing that MYC silencing significantly decreases BCR promoter activity in all of the constructs analysed (*p* < 0.0001) (Fig. 5c). Notably, deletion of PBS1 and PBS2 (1200_BCR construct) and/or deletion of PBS3 and PBS4 (Full_BCR∆34 and 1200_BCR∆34 constructs) dramatically decreases the overall promoter strength, even in presence of MYC expression, therefore confirming the critical role of these region in controlling BCR promoter activity. In line with these findings, the loss of PBS1 to 4 almost completely abrogates BCR promoter activity, suggesting that they represent core controllers of BCR promoter regulation. Interestingly, the deletion of PBS3 and PBS4 (Full_BCR∆34) induces a greater down-modulation of luciferase activity compared to PBS1 and PBS2 deletion (1200_BCR) (Full_BCR shNC 6.11 ± 0.11, Full_BCR∆34 shNC 1.86 ± 0.14, 1200_BCR shNC 3.92 ± 0.07) (Fig. 5c), in accordance with the greater ChIp enrichment observed for MYC binding at Region 2(R2) compared to Region 1(R1) (Fig. 1c). These results confirm the role of the four MYC binding sites (PBS1-4) in BCR promoter regulation, also suggesting a prominent role for PBS3 and PBS4.

## Discussion

In CML cells, after the t(9;22)(q34;q11) translocation and the formation of the Philadelphia chromosome, the BCR promoter drives the production of the BCR/ABL1 mRNA [9]. This oncogenic product is necessary and sufficient for the malignant transformation of hematopoietic cells *in-vitro* and *in-vivo* [33] and during CML progression the BCR/ABL1 oncogene persist and its level increases [34–36]. The enhanced BCR/ABL1 activity characteristic of BC phase, seems to influence proliferation, survival, genetic instability and differentiation of myeloid progenitors [3, 5–8].

A number of previous reports suggested the involvement of MYC in the clonal evolution of CML. MYC protein collaborate with BCR/ABL1 to induce blastic transformation, as assessed by the therapeutic efficacy of a silencing combination therapy for MYC and BCR/ABL1 of primary CML cells in SCID mice [37]. Interestingly, MYC is also often upregulated in BC through chromosome 8 trisomy or gene amplification [19, 20, 28, 38]. It has also been suggested that BCR can decrease MYC activity by regulating its stability at protein level [27]: BCR disruption during BCR/ABL1 translocation can thus contribute to CML transformation leading to MYC upregulation. MYC levels are also controlled by BCR/ABL1 oncoprotein at transcriptional and protein levels [22–26]. The role of MYC activity in blastic transformation is also strengthen by the fact that MYC is a known beta-catenin target gene, which has been shown to be activated in BC patients [4, 39].

According to previous reports, BCR is physiologically down-regulated upon myeloid maturation from hematopoietic stem cells (HSCs) to common myeloid progenitors (CMPs) and granulocyte-macrophage progenitors (GMPs) and this mechanism is conserved in healthy donors and in CP-CML [39]. Conversely, in BC this regulation is impaired for both BCR/ABL1 and BCR, which suggests the presence of ‘*in trans*’ deregulated transcription of both BCR and BCR/ABL1 promoters associated with CML progression [10]. A direct effect of MYC leading to an increase in BCR/ABL1 levels has recently been suggested [29], but the molecular mechanism responsible for this phenomenon has not been clarified.

We showed here that MYC/MAX heterodimer can bind to BCR promoter at four specific loci (Fig. 1), thus regulating BCR and BCR/ABL1 expression in several CML cell line models (Figs. 2 and 3). By using a luciferase reporter assay we confirmed that MYC can modulate BCR and BCR/ABL1 expression by directly controlling BCR promoter activity (Fig. 5). Interestingly, when PBS3 and PBS4 were deleted from the BCR promoter construct (Full_BCR ∆3,4; 1200_BCR ∆3,4), a dramatic decrease in luciferase activity was observed when compared to all the other constructs, thus suggesting a critical role of these two regions in the regulation of BCR promoter activity (Fig. 5).

Our results show the existence of a positive feedback mechanism between the fusion protein BCR/ABL1 and the MYC transcription factor. BCR/ABL1 expression increases MYC activity which, in turn, is able to up-regulate BCR/ABL1 levels through direct binding on BCR promoter.

## Conclusions

We present here the first description of a new pathway which places BCR and BCR/ABL1 under the transcriptional control of the MYC/MAX heterodimer. Our findings confirm the oncogenic role of MYC in CML pathogenesis, suggesting a causal relationship with the increased BCR/ABL1 levels observed during CML progression. Further analyses will be required to assess the importance of this pathway in blast crisis transformation.

## Methods

### Cell lines

The BCR/ABL-positive CML cell lines K562, LAMA-84 and KCL-22 (DSMZ, Braunschweig, Germany) were cultured in RPMI-1640 medium supplemented with 10 % FBS, 2mM L-glutamine, 100 U/ml penicillin G, 80 μg/ml gentamicin and 20 mM HEPES in a 5 % CO_2_ incubator at 37°C. The 293-FT (R700-07, Life Technologies-Thermo Fisher Scientific, Waltham, MA USA) and the 293 cell lines (R750-07, Life Technologies-Thermo Fisher Scientific) were maintained following manufacturer instructions.

### *In silico* analysis

To define the transcription factors involved in the regulation of BCR expression, *in silico* analysis of BCR promoter (1443bp from the ATG site) was done using the open access Jaspar core database [40], available on http://jaspar.genereg.net/. The putative binding sites for transcription factors were generated from the distributions of bases at each position of all the transcription factors frequency matrices available in the JASPAR database. The putative binding sites with a relative profile threshold ≥ 80 % and a binding score >8 were selected for subsequent analyses.

### Chromatin Immunoprecipitation (ChIP)

Chromatin immunoprecipitation was performed on K562 cells as previously reported [41]. Briefly, proteins were cross-linked with 0.4 % formaldehyde and cells lysed. Chromatin was fragmented with a Bioruptor sonicator system (Diagenode, SA, USA) and subsequently immunoprecipitated with anti-MYC[N-262] (SC-764X, Santa Cruz Biotechnology Inc., Texas, USA), anti-MAX[C-17] (SC-197X, Santa Cruz Biotechnology) or non-specific IgG antibodies. After purification, immunoprecipitated DNA was amplified with RT-qPCR as described in [42]. Results were visualized after separating PCR products by agarose gel with ethidium bromide staining. Primers used for the quantitative RT-PCR were: BCR-MycMax-Fw _5′_GGGAAGTTCTGAGTCAGGTCG_3′_ and BCR-MycMax-Rw _5′_TGAGTAACCAATGCCAGCACCC_3′_ for the amplification of a region including PBS1 and PBS2; 2BCR-MycMax-Fw _5′_GAGGACTGCTGCGAGTTCTGCC_3′_ and 2BCR-MycMax-Rw _5′_-GACTCCCTGGTCCATAAAGACC_3′_ for the amplification of a region including PBS3 and PBS4; NPM1_MycFw _5′_CTCGTGAGCCAGGGATGCT_3′_ and NPM1_MycRw _5′_CCCTAGTGCTACCAGCCTCTTAAC_3′_ for the amplification of MYC binding positive control; MARS-MaxFw _5′_AAGTGCGACTTGCCCTAAAA_3′_ and MARS-MaxRw _5′_CCATGCAGCTGGGACTACA_3′_ for the amplification of MAX binding positive control [31, 32].

### Real-time quantitative PCR (RT-qPCR)

Total RNA was extracted by Trizol reagent following standard procedures (Life Technologies). cDNA was synthesized from 1μg of total RNA, using Reverse Transcription Reagents (Applied Biosystems-Life Technologies). The total RNA obtained from transfectant cells were pretreated with DNAseI (Life Technologies) to avoid contamination from genomic DNA. RT-qPCR was performed using TaqMan® Brilliant II QPCR Master Mix (Agilent technologies, Santa Clara, CA, USA) for TaqMan assays or with Brilliant III Ultra-fast SYBR Green QPCR Master Mix (Agilent technologies) for SYBR Green assays on a Stratagene-MX3005P (Agilent technologies) under standard conditions. All the RT-qPCR experiments were performed in triplicate. The housekeeping gene β-glucronidase (GUSB) was used as an internal reference as assessed by [43]. For MYC and MAX expression analysis we used TaqMan® Gene Expression Assays(Applied Biosystems-Life Technologies) consisting of a pair of unlabeled PCR primers and a TaqMan® probe. TaqMan RT-qPCR was performed according to the manufacturer’s specifications. The assay identification numbers were as follows: MYC-Hs00153408_m1, MAX-Hs01105524_g1. BCR, BCR/ABL and GUSb RT-qPCR primers/probes have been previously described [10].

### Western blot analysis

Western Blot was performed as previously described [42] using the following antibodies**:** rabbit anti-MAX [C-17] (sc-197X)(Santa Cruz Biotechnology), mouse anti c-MYC[9E10] (sc-40)(Santa Cruz Biotechnology), rabbit anti-c-ABL[K-12] (sc-131)(Santa Cruz Biotechnology), rabbit anti PARP-1[E102] (ab32138)(Abcam, Cambridge, UK) and anti-ACTIN (A2066)(Sigma-Aldrich, St Louis, MO, USA).

### BCR promoter constructs and site-direct mutagenesis

pGL3_BCR promoter constructs were obtained as in [10]. Briefly, a region of 1443bp of the human BCR gene (NC_000022.11) from the first nucleotide upstream the ATG starting site was inserted in the pGL3 vector (Fig. 1b). Mutations in the BCR promoter at the MYC:MAX binding sites were introduced by the following protocol. Specific primers were designed and used to mutagenize the pGL3-Full_BCR and the pGL3-1200_BCR plasmids by using the Pfu Ultra High Fidelity enzyme (Agilent Technologies, Santa Clara, CA, USA). The products were then digested with DpnI (Roche, Indianapolis, IN, USA) and 2 μl were used to transform the competent TOP10 bacterial strain (Life Technologies). The presence of the deletion was confirmed by Sanger sequencing. The primer sequences used for mutagenesis reaction were as follows: MycMax-34-Fw _5′_GAGGTAGGTGGTGGGGCTTGGCTGTTCCAGGACTGCAGGACTG_3′_; MycMax-34-Rw _5′_CAGTCCTGCAGTCCTGGAACAGCCAAGCCCCACCACCTACCTC_3′._

### RNA interference

MYC silencing was generated infecting K562, LAMA-84 and KCL-22 cells with lentiviral particles obtained from modified FUGW lentiviral vectors: FUGW-MYC-shRNA(shMYC) kindly provided from Dr. RN Eisenman [44]**,** FUGW-H1-scrambled control shRNA (shNC) was a gift from Sally Temple [45] (Addgene plasmid # 40625; Cambridge, MA, USA). Lentiviruses were packaged in 293-FT cells by co-transfecting the shRNA vectors with the packaging pCMV-dR8.91 and VSVG plasmids using jetPRIME Polyplus (Polyplus-transfection S.A, New-York, NY, USA) following manufacturer recommendations. Lentiviruses-infected cells were analyzed for GFP positivity with a a FACSAria flow cytometer (BD Bioscience, San Jose, CA, USA) and FACS-sorted when infection efficiency was lower than 85 %.

### Generation of MYC and MAX expressing cell lines

MAX cDNA was obtained from total RNA of K562 cell line. Two primers spanning the whole MAX coding sequence (NM_145112.2) and introducing artificial HindIII and EcoRI sites at the 5′ and 3′ ends of the coding region were used for amplification and the PCR product was cloned into the pCDNA3 vector. Sequences of the primers were: MAX_Fw _5′_AATAAAGCTTGAAATGAGCGATAACGATGAC_3′_; Max_Rw _3′_AATAGAATTCCCCGAGTGGCTTAGCTGGCCT_5′._ pWZL_Blast_MYC vector was a gift from William Hahn [46] (Addgene, plasmid # 10674). K562 cells were electroporated using a GenePulser XCell (Bio-Rad, Hercules, CA) (270 V, 975 μF). Transfected cells were selected with 5 μg/ml blasticidin and/or 1 mg/ml geneticin.

### Luciferase assay

293 cells were infected with the lentiviral vectors encoding either MYC-shRNA(shMYC) or the scrambled-shRNA(shNC). RT-qPCR and Western Blot were used to confirm MYC down-regulation. Infected 293 cells were then co-transfected with pRL and pGL3/BCR promoter constructs prepared as described in Marega et al. [10] and luciferase activity was determined after 72 h using the Dual-Luciferase Reporter Assay (Promega, Madison, WI, USA) and measured with the 1450 Wallac MicroBeta®luminometer (PerkinElmer, Waltham, Massachusetts, USA). All the experiments were performed in triplicates.

### Cell viability assay

Cell viability was monitored by CellTiter 96 AQ One Solution Cell Proliferation Assay (Promega). The MTS tetrazolium reagent was added to the cells after 24h, 48h, 72h and 96h from seeding. Absorbance was assayed with a Wallac 1450 MicroBeta Trilux (PerkinElmer).

### Cell cycle analysis

Cells were fixed in ethanol and stained with propidium-iodide (Sigma-Aldrich). Flow cytometry was performed on a Becton Dickinson FACSCalibur (Becton Dickinson Immunocytometry Systems, Mountain view, CA, USA) and data were analysed by FCS Express 4 Flow Research Edition software.

### Statistical analysis

All the statistical analyses (unpaired two-tailed *T*-test) were performed by the GraphPad Prism (GraphPad, CA, USA) statistical package.

## Abbreviations

CML: Chronic Myeloid Leukaemia
BC: Blast Crisis
CP: Chronic Phase
RT-qPCR: Real-Time quantitative PCR
ChIP: Chromatin Immunoprecipitation
shNC: scrambled control RNA, negative control
